# In Vivo Cell Migration and Growth Within Electrospun Porous Nanofibrous Scaffolds with Different Pore Sizes in a Mouse Pouch Model

**DOI:** 10.3390/jfb16050181

**Published:** 2025-05-14

**Authors:** David C. Markel, Therese Bou-Akl, Bin Wu, Pawla Pawlitz, Xiaowei Yu, Liang Chen, Tong Shi, Weiping Ren

**Affiliations:** 1 Department of Orthopedics, Henry Ford Providence Southfield Hospital, Southfield, MI 48075, USA; david.markel@thecoreinstitute.com (D.C.M.); binwu777@gmail.com (B.W.); ppawlit1@hfhs.org (P.P.); wren1952@gmail.com (W.R.); 2 Department of Biomedical Engineering, Wayne State University, Detroit, MI 48201, USA; liangchen89@gmail.com (L.C.); ak5905@wayne.edu (T.S.); 3 The Core Institute, Novi, MI 48374, USA; 4 Department of Orthopedics, 6th People’s Hospital, School of Medicine, Shanghai Jiao Tong University, Shanghai 200233, China; yuxw@sjtu.edu.cn; 5 Virotech Co., Inc., Troy, MI 48085, USA

**Keywords:** porous nanofiber, electrospinning, microstructures, tissue growth, mouse pouch model, regenerative medicine

## Abstract

Cellular infiltration into traditional electrospun nanofibers (NFs) is limited due to their dense structures. We were able to obtain polycaprolactone (PCL) NFs with variable and defined pore sizes and thicknesses by using a customized programmed NF collector that controls the moving speed during electrospinning. NFs obtained by this method were tested in vitro and have shown better cell proliferation within the NFs with larger pore sizes. This study investigated in vivo host cell migration and neovascularization within implanted porous PCL NF discs using a mouse pouch model. Four types of PCL NFs were prepared and classified based on the electrospinning speed: NF-zero (static control), NF-low (0.085 mm/min), NF-mid (0.158 mm/min) and NF-high (0.232 mm/min) groups. With the increase in the speed, we observed an increase in the pore area; NF-zero (11.6 ± 6.2 μm^2^), NF-low (37.4 ± 28.6 μm^2^), NF-mid (67.6 ± 54.8 μm^2^), and NF-high (292.3 ± 286.5 μm^2^) groups. The NFs were implanted into air pouches of BALB/cJ mice. Mice without NFs served as control. Animals were sacrificed at 7 and 28 days after the implantation. Pouch tissues with implanted NFs were collected for histology (*n* = three per group and time point). The efficiency of the tissue penetration into PCL NF sheets was closely linked to the pore size and area. NFs with the highest pore area had more efficient tissue migration and new blood vessel formation compared to those with a smaller pore area. No newly formed blood vessels were observed in NF-zero sheets up to 28 days. We believe that a porous NF scaffold with a controllable pore size and thickness has great potential for tissue repair/regeneration and for other healthcare applications.

## 1. Introduction

Implant coating to improve cellular implant interaction is gaining more attention [1]. Electrospinning technology has been used extensively to produce fibrous biomaterials with characteristics similar to the native fibrous component of bones [2]. The main fibrillar matrix protein found in bone ECM is collagen with a nanoscale diameter [3]. Electrospun nanofibers are promising for many tissue engineering applications due to their high surface area, high mass to volume ratio, and small inter-fibrous pore size with high porosity [4]. The traditional electrospinning process usually produces two-dimensional (2D) and dense fibrous mats with a small pore size (<100 μm) and poor interconnectivity, which inhibits cell infiltration and proliferation [5]. Certain cell types have preferences to specific pore sizes; for example, PC12 neuronal cells showed neurite outgrowth on aligned porous electrospun nanofibers (100 nm pores) made of poly L-lactic acid and coated with polypyrrole [6]. The bone cell penetration and vascularization of porous implant materials have been shown to improve in materials with a pore size larger than 300 μm [7,8], while fibroblasts grow better in denser areas with pore sizes of 6 to 20 μm [9,10]. Electrospun nanofibrous matrices with a 3D structure and controllable pore sizes are highly desirable to promote cell infiltration, vascularization, and bone regeneration [11,12]. Electrospun nanofibers find many applications in healthcare. A potential application is a dermal substitute. Electrospun NFs made of a blend of Chitosan and PVA were evaluated by Sundaramurthi et.al, and the resultant C-PVA nanofibers showed that 3T3 fibroblast cells adhered to and proliferated on the surface of the NFs in vitro; the same material was tested in a rat model with the addition of growth factor R Spondin1, which showed an enhancement in wound healing [13]. Another application that is gaining a lot of interest is the use of electrospun NFs as local drug delivery systems to improve efficacy and decrease the side effect of the incorporated drug [14,15], or to provide a mechanical occlusion and drug delivery like the transdermal patches for hemorrhage control [16]. Electrospun NFs have been shown to improve cell migration and bone healing in vivo; in a study performed by Schofer et.al, the authors investigated the impact of electrospun PLLA NFs with and without the addition of bone morphogenetic protein 2 (BMP-2) on bone formation using a critical size rat calvarial defect model [12]. They found that PLLA NFs facilitated the filling of the bone defect, and the combined nanofibers increased the bone regeneration [12].

Many efforts were made to increase the pore size and/or modify the geometry/thickness of electrospun NFs to enhance their mechanical properties and improve the tissue regeneration caused by efficient cellular attachment and proliferation [3,17,18]. Lee et al. were able to enhance the mechanical properties and control the pore size (200–327 um) of their hybrid scaffold by combining a natural (collagen) and a synthetic polymer (PCL); this combination promoted the proliferation of the osteoblasts-like cells (MG63) [19]. A review by Teo et al. elaborated on the effect of modifying the magnetic field and the electric force on the spinning jet on the formation of 3D NFs with a specific geometry [20]. Additionally, the pore size of NFs can be increased by combining soluble and insoluble materials during electrospinning [21,22]. Phipps et al. were able to increase the pore size of their PCL/col I/nanoHA scaffolds by modifying the NF collector plate to reduce the fiber packing density during electrospinning and including a water-soluble polymer PEO. They reported that this approach was the most effective [22]. Also, methods like salt leaching [23], ice crystals [24], and photopatterning [25] have been investigated to modify NF porosity. However, the required multiple fabrication steps for a complete solvent evaporation in freeze-drying cycles remain a critical concern [26]. Yokoyama et al. were able to control the porosity of their Polyglycolic acid (PGA) nanofibers by adding wet spinning and freeze drying to the conventional electrospinning method [26]. Also, the pore size increase can be achieved by modifying the collector surface using different patterns [27,28]. Mass production can be accomplished using rolling or stacking collectors [29,30,31] and yarn [32]. However, most of these NFs are randomly wrapped and lacking the desired porosity, pore size, pore shape, geometry, and thickness that are essential for some tissue regeneration processes, like bone tissue regeneration. Moreover, these techniques are time consuming and expensive, which greatly limit their intended application.

We previously developed a corona discharge-based technology that allowed the continuous collection of PCL NF sheets on a collector surface with movable needles [33]. The working mechanism of this model is comparable to the plasma discharge phenomenon described by Huang et al. [34].

The main limitation of the prior corona discharge-based electrospun NF preparation was that the porosity was not controllable and reproducible because of the manual movement of the randomly mounted needles [33]. In addition, it was critical for us to establish a reference NF scaffold with a consistent microstructure for various applications in biomedical engineering [35]. To address this concern, we recently created four programmed collectors to obtain four types of NFs with precise pore sizes, volumes, and thicknesses by controlling their moving speed during electrospinning [36]. We found that the pore sizes of NFs obtained by this method resemble the ECM structure and porosity [36]. The effect of the various porosities on cell penetration and growth was assessed in vitro using three cell types: pre-osteoblastic MC3T3 cells, pre-osteoclastic RAW cells, and rat bone marrow stem cells [36]. The proliferation and differentiation of both MC3T3 cells and rat bone marrow stem cells were the highest on the NF-high sheets as compared to other groups (*p* < 0.01). RAW cells had the highest cell growth and differentiation on NF-mid sheets. The detailed mechanisms behind these differences are worthy of further investigation in vivo.

This study was designed as a continuation of the in vitro work, with the aim of investigating the in vivo host cell adhesion, migration, and growth into porous NF sheets with a well-defined pore size using a mouse air pouch model. We propose that the host cell migration and growth within the inner pores of the 3D NF sheets prepared by this method would be more prominent in those with a larger pore size.

## 2. Materials and Methods

### 2.1. Materials

The chemicals used in this experiment were of analytical grade and, unless specified otherwise, were procured from Sigma-Aldrich (St. Louis, MO, USA). Specifically, polycaprolactone (PCL), with a molecular weight of 70–90 KDa, along with chloroform, dimethyl sulfoxide (DMSO), and dimethylformamide (DMF), were sourced from Sigma-Aldrich (St. Louis, MO, USA). Female BALB/cJ mice, 12 weeks old and averaging 20 g in weight, were purchased from Charles River Laboratories (Wilmington, MA, USA).

### 2.2. Electrospun PCL NF Sheets Preparation

**Figure 1:**
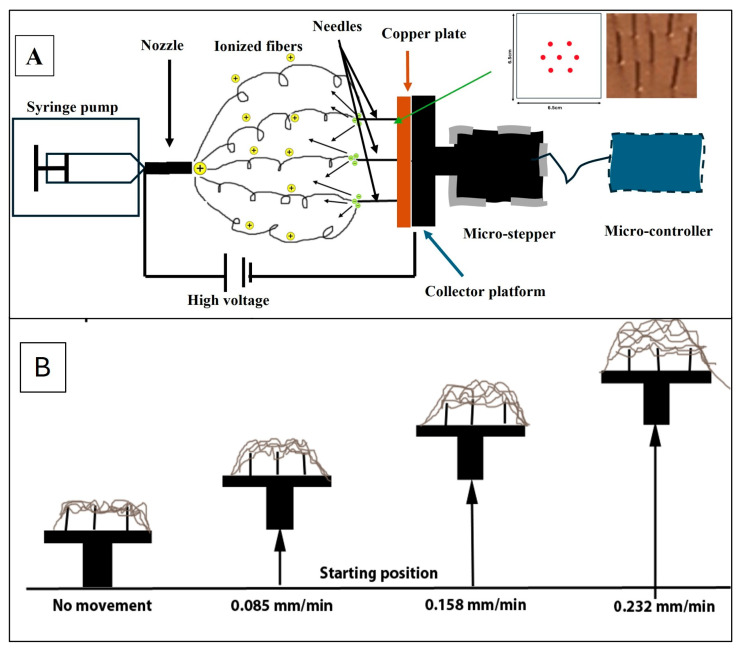
A schematic of the electrospinning process. (**A**) The components of the collection device include a microcontroller, stepper motor, and collector surface lined with a copper plate and outfitted with needles arranged in a hexagonal configuration (inset). (**B**) Schematic of the electrospun structure and showing the speed of the stepper.

**Figure 2:**
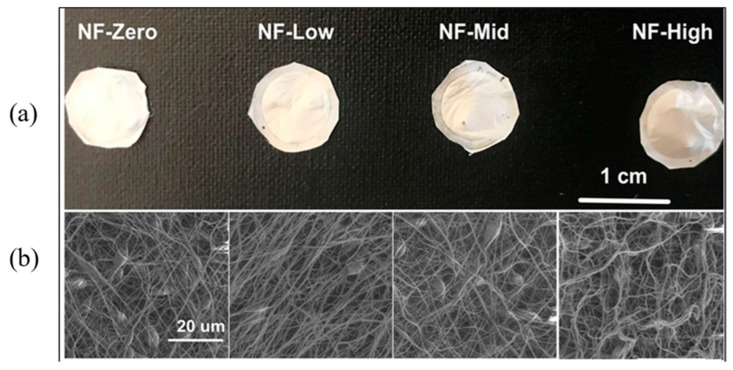
(**a**) Images of 3D PCL NFs formed at defined collector moving speed. NF-zero (0 mm/min), NF-low (0.085 mm/min), NF-mid (0.158 mm/min) and NF-high (0.232 mm/min) sheets. (**b**) SEM morphology of 3D NF sheets (magnification ×2000).

The NF sheets used for this work were prepared using a custom-made collector apparatus comprised of four collector platforms, and each one is driven by an individual stepper motor driver controlled by Arduino controller units, allowing for synchronized yet independent functioning. The platforms are equipped with seven grounded needles, each with a diameter of 0.18 mm, arranged in a hexagonal layout (Figure 1A), which ensures a balanced distribution of electrical charges. This configuration promotes a uniform deposition of NFs across the platform [36]. This prototype NF collector allows for the accurate control of the automated platform movement, which is believed to affect the microstructure of NFs deposited on the surface during electrospinning (Figure 2b). The electrospinning was performed in a small room with a consistent temperature and humidity. A humidifier and a humidity sensor were used to maintain stable humidity in the electrospinning chamber.

A PCL solution with a concentration of 11% (*w*/*v*) was prepared by dissolving PCL in an equal volume mixture (1:1) of chloroform and dimethylformamide (DMF) overnight [37]. This solution was then drawn into a 5 mL syringe fitted with a blunt 26G spinneret, which was connected to a syringe pump (R100E, Razel Scientific Instruments, St. Albans, VT, USA) set at a flow rate of 1 mL/h. A high-voltage power supply (ES40P, Gamma High Voltage Research Inc., Ormond Beach, FL, USA) was connected to the nozzle by alligator clips. Electrospinning parameters were set at 15 kV for voltage, 40% for humidity, and at 10 cm between the needle tip and the collector. The collector platforms were advanced continuously at respective speeds of 0 (NF-0 sheet), 0.085 (NF-low sheet), 0.158 (NF-mid sheet), and 0.232 (NF-high sheet) mm/min. The electrospinning process was conducted in six 10 min cycles; the generated PCL NFs were cut into discs 0.5 cm in diameter with a thickness of 2 mm and then sterilized by UV light exposure.

### 2.3. Scanning Electron Microscopy (SEM)

The generated sheets were gold-coated (Gold Sputter EFFA Coater, Redding, CA, USA), and the morphology of the NFs was characterized by scanning electron microscopy (SEM) (JSM-6510LV-LGS, Peabody, MA, USA) at a 10 kV accelerating voltage. Three NFs were prepared for each group. Three SEM images (2000×) were taken from each NF; thus, nine images for each group were prepared in total. The average pore area (mm^2^) was measured from SEM images using analysis software, version 1.51 (Image J, National Institute of Health, Bethesda, MD, USA).

### 2.4. Mouse Air Pouch Model

A mouse air pouch model is anticipated to enhance the interaction between host tissues and the implanted NF sheets, providing a crucial opportunity to deepen our understanding of the relationship between host tissue cells and NF sheets. The approval of this work was granted by the Animal Care Committee and the Laboratory Animal Administration of Ascension Providence Hospital. This study involved thirty female BALB/cJ mice, assigned randomly into five groups, with three mice per group at each time point (Table 1). Air pouches were created by a subcutaneous injection of 1.5 mL of sterile air into the dorsal area (interscapular region) 2–3 times over a six-day period, to allow the pouches to mature [38]. The dorsal area of the mouse was chosen for the pouch location because it is well away from any vital organs, has muscle tissue in the vicinity, and it is difficult for the animal to reach and/or scratch. Under anesthesia (Ketamine and Xylazine), sterile PCL NF discs, varying in pore size, were placed in the matured pouches via a 5 mm incision. Control mice, which did not receive PCL NF implants, were included for comparison. The animals were euthanized at 7 and 28 days after implantation, and the pouch tissues containing the NFs were collected for histological analysis.

### 2.5. Histological Analysis

The collected pouch tissues were preserved in 10% formaldehyde and subsequently processed for histology using paraffin embedding. Five μm sections were stained with hematoxylin and eosin (H&E). The stained slides were scanned using Pathscan Pro slide scanner at 4× magnification and the details of cell infiltration within the NFs were examined under a light microscope.

### 2.6. Statistical Analysis

The statistical significance of the results was determined using ANOVA for differences among multiple groups, and *p* < 0.05 was considered statistically significant.

## 3. Results and Discussion

A programmed NF collector platform was developed and allowed for the ability to obtain porous NFs with desired pore sizes in a guided and reproducible method by setting the moving speed of each collector segment and using a unique hexagonal needle array design (Figure 1A) [36]. This technology led us to the one-step preparation of homogenous porous NF sheets with the desired microstructure and thickness, without any delamination or folding. We demonstrated in vitro that the evaluated cells were sensitive to the NFs with different pore sizes, and the response was cell-specific [36].

The hydrophobic PCL polymer was selected for the preparation of the NFs sheets used in this work because of its known biodegradability, biocompatibility, and viscoelastic properties [37,39,40]. Photos of the 3D PCL NF sheets with different porosities are shown in Figure 2a. The average pore area (μm^2^) of PCL NF sheets obtained by the current method was proportionally increased with the increase in the NF collector speed. The NF-high sheets had the largest average pore area (292.3 ± 286.5 μm^2^), followed by NF-mid (67.6 ± 54.8 μm^2^), NF-low (37.4 ± 28.6 μm^2^), and NF-zero (11.6 ± 6.2 μm^2^) sheets. It is worth noting that all the nanofibers formed by the current method had a larger pore area than the 2D NFs (pore area < 0.1 μm^2^) collected by flat aluminum foil [41]. In addition, our previous work demonstrated that 8% of pores from NF-mid sheets and 34% of pores from NF-high sheets were larger than 200 μm^2^. All the pores in NF-zero sheets were less than 50 μm [36].

Cell growth and differentiation are highly dependent on the ECM structure of specific tissues [42]. Some studies have shown that a minimum pore size of 10 μm is required for fibroblast infiltration [9,43]. In addition to improved cell infiltration, NFs with a pore size ~100 μm promoted wound healing in vivo [44,45]. Pelipenko et al. [46] found that the migration of keratinocytes was significantly inhibited within NFs with a small pore size (<3 µm). Pore sizes play a critical role in cell attachment, cell-to-cell interaction, and cell–surface interaction. The authors Bružauskaitė et al. reviewed the effect of pore sizes on the final recovery of diseased organs [47]. They concluded that specific cells have specific pore size requirements, and that scaffolds with pore sizes < 1 μm can be used when investigating cell–surface interaction, while pore sizes around 1–3 μm can be used when cell–cell communication is required. They also stated that cellular adhesion was best noted on 3D scaffolds with 100 μm pores, while nerve cells and fibroblast growth was the best on scaffolds with 50–160 μm pore sizes [47]. A recent review by P. Yadav et al. highlighted the effect of pore size and porosity on the mechanical properties of scaffolds prepared by various methods for use in tissue engineering [48]. The authors also described several methods used to quantify porosity, fiber diameter, and pore size. Some of the described methods are the liquid displacement method, SEM image analysis, and mercury porosimetry and permeability [48]. Presson et al. studied the osteogenic hMSC differentiation into osteoblast cells on 2D and 3D composite fibers (224–249 μm pores) prepared from polylactic acid (PLA) and hydroxyapatite powders using the melt spinning process. They reported that the 3D woven scaffolds had a major impact on hMSC proliferation and activation [49]. We have previously shown in vitro that MC3T3 cells migrated deeper into the 3D PCL NFs with a pore size > 100 um relative to their migration into the 2D NFs with a smaller pore size [33]. We expect that fibroblasts and other inflammatory cells found in vivo would have the same behavior.

**Figure 3:**
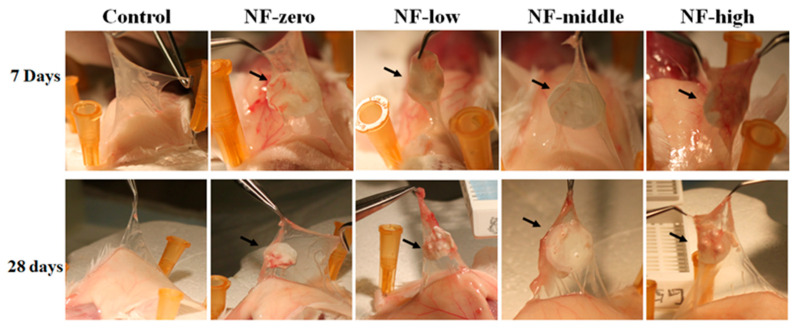
Macro morphology of pouches collected at 7 days and 28 days after implantation of 3D NFs. Arrowhead indicated the pouch with implanted 3D PCL NF sheet.

The mouse air pouch model that was initially described by Edwards et al. [50] has been widely used to evaluate the host tissue response to the implanted biomaterials under a controllable experimental condition [51,52]. Reports have demonstrated that the repeated injection of air into the subcutaneous tissue on the back of a rodent results in the formation of a blind connective tissue cavity. This model has been used by our group and by others to evaluate tissue biomaterial interactions in vivo. In this study, we implanted sterilized porous NF sheets with different pore sizes into the mice air pouches. Pouch tissues were collected at 7 days and 28 days after NF sheet implantation, respectively (*n* = three for each time points). Macroscopic photos of the pouches from all groups at the time of sacrifice are shown in Figure 3. When compared to the negative control empty pouch, no noticeable acute or chronic tissue inflammation (hyperemia and/or edema) was observed in pouches with NF implantation both 7 days and 28 days after implantation. As compared to the 7-day group, an increase in pouch thickness with implanted NFs was observed in the 28-day group, indicating increased cellular growth within the implanted NF sheets, particularly in the NF-high group.

**Figure 4:**
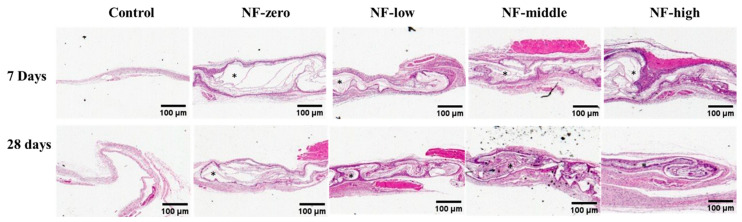
Morphology of pouches of paraffin section slides with H&E stains collected at 7 days and 28 days after implantation of 3D NFs. (Magnification ×4), scale bar 100 µm. * Indicates the pouch cavity.

**Figure 5:**
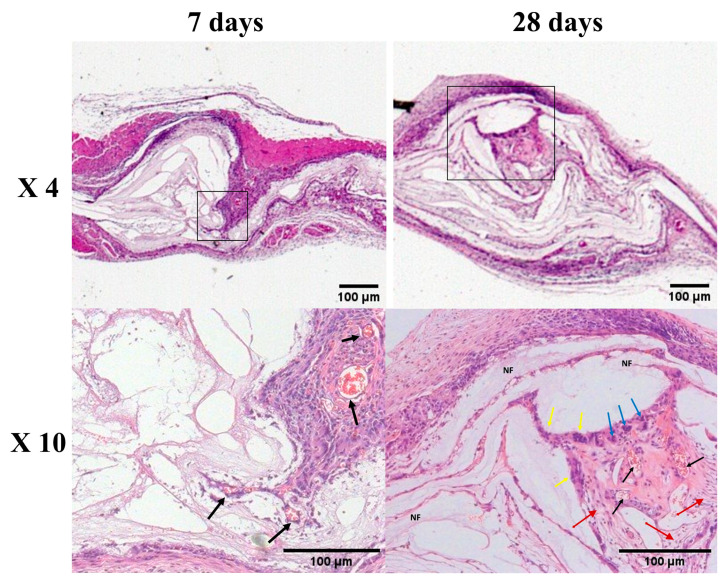
Morphology of NF-high sheets of paraffin section slides with H&E stains collected at 7 days and 28 days after implantation of NF-high 3D NFs (×4 and ×10 magnifications, respectively). Scale bar 100 µm. Box: region of interest; black arrows show the new blood vessels within projected penetrating tissues across the NF matrix. Blue arrows show some macrophages, yellow arrows show some polymorphonuclear cells, and red arrows show some fibroblasts. Nanofibers are designated by NF.

The histological analysis of H&E-stained tissue sections (Figure 4) shows that seven days after implantation, very limited cell infiltration and migration was observed in the NF-zero group, forming a thin and delicate band of uniform fibrous tissue on the surface of implanted NFs comprised mainly of fibroblasts. In contrast, an early migration and growth of the host tissue cells into the entire layer of the NF matrix in a projected front pattern was observed in the more porous NF matrices (NF-low, NF-mid, and NF-high groups), especially in NF-high group, accompanied with extensive cell growth within the NF matrix. Most of the migrated cells were predominantly fibroblasts, followed by macrophages and polymorphonuclear cells (PMNs), with sizes between 10 and 30 microns (Figure 5). A similar pattern was observed 28 days after PCL NF implantation. As compared to NF-zero group, host cell penetration and migration covering the entire layer of PCL NFs matrix was observed.

The new tissue and new blood vessel formation within the NF matrix in the NF-high group is shown in Figure 5. New blood vessels formed within the new tissues close to the NF matrix, suggesting a dynamic interaction between the implanted NF matrix with host tissue migration.

Data from the histological analysis indicated that the implanted PCL NFs were biocompatible, as manifested by the lack of a noticeable inflammatory tissue response and/or foreign body reaction compared to the NF-free control, both at 7 days and 28 days [51,52,53]. This study illustrates the host cell migration and growth in a porous PCL NF sheet with a thickness of 2 mm. Many previous in vivo studies used NF sheets with a thickness of less than 1 mm in the animal models [54,55,56].

The capability of the host cell migration and growth in a synthetic scaffold is critical for the successful repair of the defected tissues [36,57]. Several investigators have demonstrated that cell infiltration is largely determined by the porosity and pore size of the scaffolds [33,36]. Jiang et al. [58] used a rat subcutaneous implantation model to investigate the cellular infiltration within an implanted layered PCL NF scaffold. They prepared a porous 3 mm thick PCL NF scaffold using a layer-by-layer folding approach with up to 100 μm spaces between adjacent layers. Compared to traditional 2D dense NF scaffolds, progressive cell growth within the layered 3D PCL NFs up to 8 weeks was mainly reliant on the porous microstructure and the gap between the layers. In another study, Jiang et al. [59] reported that porous PCL NFs with arrayed holes significantly promoted cellular infiltration and new blood cell formation along the arrayed holes in a rat subcutaneous implantation model. Also noteworthy is that the microstructure of the thicker PCL NFs described here was homogenous and continuous, without gaps in between. This unique structure is critical for promoting tissue regeneration.

A desired porous NF matrix was needed to provide a temporal structural support for the host tissue migration and ingrowth. Data from the current animal study correlates with the findings from our previous in vitro cell culture study [33,36]. The NF-high group with the pore volume > 200 μm^2^ allowed for more cell migration into the vicinity of the material (Figure 4 and Figure 5) in comparison to the NFs with smaller pore size, and this promoted new blood vessel formation as well.

Neovascularization plays a major role in promoting tissue regeneration at the site of NF implantation by providing oxygen and nutrients to the newly migrated cells [58,60]. Beier et al. [61] were the first to report the vascularization pattern within PCL NF scaffolds after subcutaneous implantation in rats using a micro-CT-scan approach. They concluded that the specific vascularization pattern within the implanted scaffolds could determine their utility for tissue engineering applications. Jiang et al. [58] reported that no blood vessels were observed within a 2D NF scaffold due to limited cellular infiltration into the scaffold. However, new blood vessels appeared at week two and their density increased with time to reach 30 vessels mm^−2^ at week eight within a porous PCL NF scaffold. Our data showed a similar finding; new blood vessels were formed in porous PCL NFs as early as one week after implantation, especially in NFs with higher pore sizes (NF-mid and NF-high group, Figure 5). In contrast, no newly formed blood vessels were observed in the NF-zero group up to 28 days.

Some limitations of this study are as follows. In the current work, we did not quantify the fiber diameter and only focused on the pore size and pore volume; knowing that fiber diameter changes may dramatically affect the mechanical properties of the formed nanofibers, we will include that measurement in future studies. The histological analysis of host cell penetration and vascular formation were descriptive, not quantitative, due to the irregularities of the air pouches and the low sample number; quantitative evaluations for cell density and/or infiltration depth into the nanofibers will be performed in future studies using this model. Only female mice were used in the study; even though it is preferable to use both sexes in research, we do not expect to have differences related to the size or shape of the used material, which is soft and circular (5 mm in diameter). However, there may be a difference in their cellular response to the implanted material.

## 4. Conclusions

The mouse air pouch model has been widely used as a reliable model for the interaction between host tissue and implanted biomaterials. This model provides space to implant materials and generates sufficient tissue for analyses. The tissue from the pouch has advantages for the investigation of cellular infiltration and movement within an implanted material. In the current work, we demonstrated that the relationship between the PCL NFs and the host cell migration was dependent on the pore size of the NFs. Cell migration and new blood vessel formation were mostly observed within the NFs with the highest pore area (NF-high group, >200 μm^2^). We believe that porous NF scaffolds with a controllable pore size, thickness, and geometry are suitable for many tissue engineering applications.

## Figures and Tables

**Table 1:** Mouse experimental groups.

Group	*n* *	NF Discs (2 mm Thick and 0.5 cm in Diameter)
1	6	No PCL NF (control)
2	6	PCL NFs (NF-0 sheet)
3	6	PCL NFs (NF-low sheet)
4	6	PCL NFs (NF-middle sheet)
5	6	PCL NFs (NF-high sheet)

* *n* = 3 for mice sacrificed at 7 and 28 days, respectively.

## Data Availability

The raw data supporting the conclusions of this article will be made available by the authors on request.
